# Clinical significance of UGT1A1 polymorphism and expression of ERCC1, BRCA1, TYMS, RRM1, TUBB3, STMN1 and TOP2A in gastric cancer

**DOI:** 10.1186/s12876-016-0561-x

**Published:** 2017-01-05

**Authors:** Yongkuan Cao, Guohu Zhang, Peihong Wang, Jun Zhou, Wei Gan, Yaning Song, Ling Huang, Ya Zhang, Guode Luo, Jiaqing Gong, Lin Zhang

**Affiliations:** Department of Gastrointestinal Surgery, Center of General Surgery of P.L.A., Chengdu Army General Hospital, No.270 Rongdu avenue, Chengdu, 610083 Sichuan Province China

**Keywords:** Gastric cancer, Individualized treatment, TYMS, TUBB3, STMN1, UGT1A1 polymorphism

## Abstract

**Background:**

Individualized therapeutic regimen is a recently intensively pursued approach for targeting diseases, in which the search for biomarkers was considered the first and most important. Thus, the goal of this study was to investigate whether the UGT1A1, ERCC1, BRCA1, TYMS, RRM1, TUBB3, STMN1 and TOP2A genes are underlying biomarkers for gastric cancer, which, to our knowledge, has not been performed.

**Methods:**

Ninety-eight tissue specimens were collected from gastric cancer patients between May 2012 and March 2015. A multiplex branched DNA liquidchip technology was used for measuring the mRNA expressions of ERCC1, BRCA1, TYMS, RRM1, TUBB3, STMN1 and TOP2A. Direct sequencing was performed for determination of UGT1A1 polymorphisms. Furthermore, correlations between gene expressions, polymorphisms and clinicopathological characteristics were investigated.

**Results:**

The expressions of TYMS, TUBB3 and STMN1 were significantly associated with the clinicopathological characteristics of age, gender and family history of gastric cancer, but not with differentiation, growth patterns, metastasis and TNM staging in patients with gastric cancer. No clinical characteristics were correlated with the expressions of ERCC1, BRCA1, RRM1 and TOP2A. Additionally, patients carrying G allele at −211 of UGT1A1 were predisposed to developing tubular adenocarcinoma, while individuals carrying 6TAA or G allele respectively at *28 or −3156 of UGT1A1 tended to have a local invasion.

**Conclusions:**

The UGT1A1 polymorphism may be useful to screen the risk population of gastric cancer, while TYMS, TUBB3 and STMN1 may be potential biomarkers for prognosis and chemotherapy guidance.

**Electronic supplementary material:**

The online version of this article (doi:10.1186/s12876-016-0561-x) contains supplementary material, which is available to authorized users.

## Background

Gastric cancer is the second most common malignancy and the third most common cause of cancer-related death in China, with an estimated 423,500 newly diagnosed cases and 298,500 deaths in 2012 [1]. Radical surgery followed by adjuvant chemotherapy is the mostly used strategy for management of gastric cancer. Nevertheless, the overall 5-year survival rate is still below 50% [2], which may result from the insensitivity and/or tolerance to chemotherapy in some gastric cancer patients. Consequently, screening effective biomarkers has become a hot topic in the field of gastric cancer in order to develop individualized therapies and make patients obtain more beneficial effects.

Recently, several studies have suggested that the expressions of excision repair cross complementing 1 (ERCC1), breast cancer susceptibility gene breast cancer 1 (BRCA1), thymidylate synthetase (TYMS), ribonucleotide reductase M1 (RRM1), β-tubulin III (TUBB3), stathmin1 (STMN1) and topoisomerase IIα (TOP2A) genes are differential in cancer tissues and are closely associated with the clinicopathological characteristics of patients (such as clinical staging or lymphatic metastasis), which make them suitable to act as possible biomarkers to evaluate the therapeutic response and survival rates of cancer patients [3–8]. Although mounting evidence also indicates their important roles in gastric cancer [9–15], no study, to our knowledge, has been conducted to simultaneously detect their expressions in the samples of gastric cancer like other cancers [3–8].

In addition, genetic polymorphisms also possess important clinical values in predicting a susceptibility to cancer and the ability of an individual to respond to therapeutic agents. For example, UDP-glucuronosyltransferases (UGTs) are important enzymes responsible for glucuronidation of serum bilirubin, which is a natural antioxidant. The inter-individual variation in enzyme activity of UGT1A1 isozyme due to the polymorphisms at positions −3156 (G > A), 211 (G/A) and TATA Box (6TAA/7TAA) leads to the lack of bilirubin and the development of cancer [16, 17]. UGTs also can transform toxic components (chemotherapy drugs) to less or nontoxic hydro-soluble forms, resulting in the failure or resistance of chemotherapy [18]. Although there have reports to investigate the relationship between UGT1A1 polymorphisms and chemotherapy drug (irinotecan) toxicity or response [19–21], no studies have been performed to explore the association of UGT1A1 polymorphisms with clinicopathological features, which was the goal of our study.

The present study aims to detect the UGT1A1 polymorphisms and expressions of ERCC1, BRCA1, TYMS, RRM1, TUBB3, STMN1 and TOP2A in the tumor tissues from gastric cancer patients and explore their relationships with the clinicopathological characteristics, hoping to provide guidance for targeted cancer therapies individually.

## Methods

### Tissue samples

A total of 98 tissue specimens were collected from gastric cancer patients in our hospital between May 2012 and March 2015. The samples were formalin-fixed paraffin-embedded (FFPE) or fresh frozen after radical surgery. All of them were histologically confirmed by two independent, experienced pathologists. All patients had complete medical records and no patient received neoadjuvant treatments prior to the primary surgery. All patients gave written informed consents for sample retention, analysis for research purposes and paper publication. The study protocol was approved by the ethics committee of Chengdu Military General Hospital of PLA, China.

### Detection of mRNA expression levels

A multiplex branched DNA liquidchip (MBL) technology (Guangzhou SurExam Bio-Tech Co., Ltd., China) was used for quantitative determination of all the genes at the mRNA level in the tissue samples simultaneously as previously reported [3, 22, 23]. Briefly, the tissue samples were lysed in buffer at 56 °C for 2 h. The lysed product was transferred to a 96-well plate in which blocking reagent, capture beads and target gene-specific probe sets were included, and then incubated at 55 °C overnight on a shaker, followed by adding the hybridization mixture. Signals for bound target mRNA were amplified with streptavidin‑phycoerythrin at 50 °C for 30 min. The fluorescence value of each sample was recognized and analyzed by the Luminex 200 system (Luminex, Austin, TX, USA) to represent the mRNA expression level of each gene. The expression level of each gene was divided into low expression (<25%), middle expression (25 ~ 75%) and high expression (>75%) by comparing to the cut‑off value of each gene which was provided by Guangzhou Surexam Medical Test Center [24].

### UGT1A1 polymorphism analysis

DNA was extracted from tissue samples using a phenol-chloroform method. Single nucleotide polymorphisms for the TATA box of the promoter (UGT1A1*28, 6TAA/7TAA) and exon 1 (−211 G/A) were simultaneously determined with the PCR primer sequences as follows: 5’-ATTAACTTGGTGTATCGATTGG-3’ and 5’-AAGCATAGCAGAGTCCTTTTTTA-3’[25], while the PCR primer sequences for the phenobarbital responsive enhancer module region of the promoter (−3156 G/A) were 5’-CTGGGGATAAACATGGGATG-3’ and 5’- CACCACCACTTCTGGAACCT-3’ [26]. Direct sequencing was performed using the ABIPRISM310 Genetic Analyzer (Applied Biosystems, Foster City, CA) according to standard protocols.

### Statistical analysis

Statistical analyses were conducted using the SPSS v.19.0 statistical software (SPSS, Chicago, IL, USA). Statistically significant differences were evaluated by the chi-square test for categorical variables and *t*-test for continuous variables. *P* value of < 0.05 was considered statistically significant.

## Results

### Patient characteristics

The patient characteristics are summarized in Table 1 (raw data see Additional file 1). Of the 98 patients, 76 (77.6%) were males and 22 (22.4%) were females. Ages of patients ranged from 32 to 77 years, with a mean of 56.92 ± 10.15 years. Thus, patients were stratified into two age groups (age > 57 years and age < 57 years). Histologically, all 98 lesions were classified into tubular adenocarcinoma (*n* = 75) and non-tubular adenocarcinoma (including mucinous adenocarcinoma, *n* = 21; and other types, *n* = 2). Further, samples with tubular adenocarcinoma was respectively shown to be well, moderately or poorly differentiated in 1, 21 and 53 patients, while the mucinous adenocarcinoma included poor differentiation and un-differentiation in 6 and 15 patients, respectively. Metastasis, including lymph node and distant involvement, was present in 55 (56.1%) patients. Approximately 81% (79/98) of the patients were diagnosed with a three- or four-level invasion depth. According to the 2002 AJCC TNM staging criteria [27], patients were classified as stage I (18, 18.4%), II (29, 29.6%), III (39, 39.8%) and IV (12, 12.2%) tumor. Thirty-two patients had the history of alcohol intake and most of the patients (93%) did not have the family history of gastric cancer.

**Table 1:** Characteristics of 98 gastric cancer patients

Characteristic	Cases, *n* (%)
Gender	
Male	76 (77.6)
Female	22 (22.4)
Age	
< 57 years	41 (41.8)
> 57 years	57 (58.2)
Family history	
Yes	5 (5.1)
No	93 (94.9)
Alcohol history	
Yes	32 (32.7)
No	66 (67.3)
Histological type	
Tubular adenocarcinoma	75 (76.5)
Non-tubular adenocarcinoma	23 (23.5)
Differentiation	
Undifferentiated	15 (15.3)
Poor	60 (61.2)
Moderate	22 (22.4)
Well	1 (1.0)
Growth patterns	
Expansive	2 (2.0)
Invasive	71 (72.4)
Expansive + invasive	25 (25.5)
Depth of invasion	
T1	14 (14.3)
T2	5 (5.1)
T3	3 (3.1)
T4	76 (77.6)
Lymph node metastasis	
N0	52 (53.1)
N1	32 (32.7)
N2	11 (11.2)
Undetermined	3 (3.1)
Distant metastasis	
M0	86 (87.8)
M1	12 (12.2)
TNM staging	
I	18 (18.4)
IIA-IIC	29 (29.6)
IIIA-IIIC	39 (39.8)
IVA-IVB	12 (12.2)

### Gene expression and their relationships with clinical characteristics

There were no significant associations between all 12 clinicopathological characteristics and the expressions of ERCC1, BRCA1, RRM1 and TOP2A. The expressions of TYMS (Table 2), TUBB3 and STMN1 (Table 3) were found to be correlated with age, in which patients with age < 57 years exhibited lower expressions of TYMS (*P* = 0.044), TUBB3 (*P* = 0.024) and STMN1 (*P* = 0.042). Furthermore, lower expression of TUBB3 was observed in female patients (*P* = 0.026) and patients having a family history of gastric cancer (*P* = 0.025) (Table 3). Similar to ERCC1, BRCA1, RRM1 and TOP2A, no correlations were also seen between clinicopathological characteristics of differentiation, growth patterns, metastasis and TNM staging, and the expression levels of TYMS, TUBB3 and STMN1 (Tables 2 and 3).

**Table 2:** Relationships between gene expression and clinical characteristics

Clinical features	ERCC1			BRCA1			TYMS			RRM1		
	Low	Middle	High	Low	Middle	High	Low	Middle	High	Low	Middle	High
Gender												
Male	11 (14.5)	46 (60.5)	19 (25.0)	6 (7.9)	39 (51.3)	31 (40.8)	24 (31.6)	36 (47.4)	16 (21.1)	45 (31.6)	25 (31.6)	6 (31.6)
Female	6 (27.3)	8 (36.4)	8 (36.4)	3 (13.6)	11 (50.0)	8 (36.4)	6 (27.3)	9 (40.9)	7 (31.8)	7 (31.6)	11 (31.6)	4 (31.6)
*χ* ^2^, *P*	4.219, .219			0.702, 0.704			1.101, 0.577			5.541, 0.063		
Age												
< 57 years	7 (17.1)	19 (46.3)	15 (36.6)	6 (14.6)	22 (53.7)	13 (31.7)	18 (43.9)	14 (34.1)	9 (22.0)	20 (48.8)	19 (46.3)	2 (4.9)
> 57 years	10 (17.5)	35 (61.4)	12 (21.1)	3 (5.3)	28 (49.1)	26 (45.6)	12 (21.1)	31 (54.4)	14 (24.6)	32 (56.1)	17 (29.8)	8 (14.0)
*χ* ^2^, *P*	3.073, 0.215			3.535, 0.171			6.264, **0.044**			3.974, 0.137		
Family history												
Yes	0 (0.0)	3 (60.0)	2 (40.0)	1 (20.0)	3 (60.0)	1 (20.0)	2 (40.0)	2 (40.0)	1 (20.0)	2 (40.0)	3 (60.0)	0 (0.0)
No	17 (18.3)	51 (54.8)	25 (26.9)	8 (8.6)	47 (50.5)	38 (40.9)	28 (30.1)	43 (46.2)	22 (23.7)	50 (53.8)	33 (35.5)	10 (10.8)
*χ* ^2^, *P*	2.065, 0.356			1.219, 0.544			0.209, 0.901			1.889, 0.389		
Alcohol history												
Yes	6 (18.8)	18 (56.3)	8 (25.0)	2 (6.3)	15 (46.9)	15 (46.9)	7 (21.9)	17 (53.1)	8 (25.0)	15 (46.9)	14 (43.8)	3 (9.4)
No	11 (16.7)	36 (54.5)	19 (28.8)	7 (10.6)	35 (53.0)	24 (36.4)	23 (34.8)	28 (42.4)	15 (22.7)	37 (56.1)	22 (33.3)	7 (10.6)
*χ* ^2^, *P*	0.178, 0.915			1.204, 0.548			1.770, 0.413			1.011, 0.603		
Histological type												
Tubular adenocarcinoma	14 (18.7)	40 (53.3)	21 (28.0)	6 (8.0)	37 (49.3)	32 (42.7)	20 (26.7)	36 (48.0)	19 (25.3)	40 (53.3)	26 (34.7)	9 (12.0)
Non-tubular adenocarcinoma	3 (13.0)	14 (60.9)	6 (26.1)	3 (13.0)	13 (56.5)	7 (30.4)	10 (43.5)	9 (39.1)	4 (17.4)	12 (52.2)	10 (43.5)	1 (4.3)
*χ* ^2^, *P*	0.526, 0.769			1.328, 0.515			2.400, 0.301			1.387, 0.500		
Differentiation												
Undifferentiated	2 (13.3)	8 (53.3)	5 (33.3)	2 (13.3)	8 (53.3)	5 (33.3)	7 (46.7)	5 (33.3)	3 (20.0)	7 (46.7)	8 (53.3)	0 (0.0)
Poor	10 (16.7)	33 (55.0)	17 (28.3)	4 (6.7)	32 (53.3)	24 (40.0)	15 (25.0)	31 (51.7)	14 (23.3)	31 (51.7)	23 (38.3)	6 (10.0)
Moderate	4 (18.2)	13 (59.1)	5 (22.7)	3 (13.6)	10 (45.5)	9 (40.9)	8 (36.4)	9 (40.9)	5 (22.7)	14 (63.6)	5 (22.7)	3 (13.6)
Well	1 (100.0)	0 (0.0)	0 (0.0)	0 (0.0)	0 (0.0)	1 (100.0)	0 (0.0)	0 (0.0)	1 (100.0)	0 (0.0)	0 (0.0)	1 (100.0)
*χ* ^2^, *P*	4.132, 0.659			3.350, 0.764			6.067, 0.416			10.825, 0.094		
Growth patterns												
Expansive	0 (0.0)	0 (0.0)	2 (100.0)	0 (0.0)	0 (0.0)	2 (100.0)	2 (100.0)	0 (0.0)	0 (0.0)	1 (50.0)	1 (50.0)	0 (0.0)
Invasive	11 (15.5)	41 (57.7)	19 (26.8)	7 (9.9)	34 (47.9)	30 (42.3)	18 (25.4)	33 (46.5)	20 (28.2)	34 (47.9)	30 (42.3)	7 (9.9)
Expansive + invasive	6 (24.0)	13 (52.0)	6 (24.0)	2 (8.0)	16 (64.0)	7 (28.0)	10 (40.0)	12 (48.0)	3 (12.0)	17 (68.0)	5 (20.0)	3 (12.0)
*χ* ^2^, *P*	6.141	0.189		5.744	0.219		8.438	0.077		4.765	0.312	
Depth of invasion												
T1	2 (14.3)	5 (35.7)	7 (50.0)	1 (7.1)	8 (57.1)	5 (35.7)	7 (50.0)	4 (28.6)	3 (21.4)	9 (64.3)	4 (28.6)	1 (7.1)
T2	2 (40.0)	2 (40.0)	1 (20.0)	0 (0.0)	2 (40.0)	3 (60.0)	2 (40.0)	2 (40.0)	1 (20.0)	2 (40.0)	2 (40.0)	1 (20.0)
T3	1 (33.3)	2 (66.6)	0 (0.0)	0 (0.0)	0 (0.0)	3 (100.0)	0 (0.0)	1 (33.3)	2 (66.6)	2 (66.6)	0 (0.0)	1 (33.3)
T4	12 (15.8)	45 (59.2)	19 (25.0)	8 (10.5)	40 (52.6)	28 (36.8)	21 (27.6)	38 (50.0)	17 (22.4)	39 (51.3)	30 (39.5)	7 (9.2)
*χ* ^2^, *P*	7.241	0.299		7.599	0.269		6.797	0.340		4.839	0.621	
Lymph node metastasis												
N0	10 (19.2)	28 (53.8)	14 (26.9)	3 (5.8)	30 (57.7)	19 (36.5)	18 (34.6)	22 (42.3)	12 (23.1)	29 (55.8)	19 (36.5)	4 (7.7)
N1	5 (15.6)	18 (56.3)	9 (28.1)	3 (9.4)	14 (43.8)	15 (46.9)	8 (25.0)	15 (46.9)	9 (28.1)	14 (43.8)	15 (46.9)	3 (9.4)
N2	1 (9.1)	7 (63.6)	3 (27.3)	2 (18.2)	6 (54.5)	3 (27.3)	3 (27.3)	7 (63.6)	1 (9.1)	6 (54.5)	2 (18.2)	3 (27.3)
Undetermined	1 (33.3)	1 (33.3)	1 (33.3)	1 (33.3)	0 (0.0)	2 (66.7)	1 (33.3)	1 (33.3)	1 (33.3)	3 (100.0)	0 (0.0)	0 (0.0)
*χ* ^2^, *P*	1.538	0.957		7.979	0.240		3.262	0.775		9.043	0.172	
Distant metastasis												
M0	15 (17.4)	48 (55.8)	23 (26.7)	7 (8.1)	45 (52.3)	34 (39.5)	28 (32.6)	38 (44.2)	20 (23.3)	44 (51.2)	33 (38.4)	9 (10.5)
M1	2 (16.7)	6 (50.0)	4 (33.3)	2 (16.7)	5 (41.7)	5 (41.7)	2 (16.7)	7 (58.3)	3 (25.0)	8 (66.7)	3 (25.0)	1 (8.3)
*χ* ^2^, *P*	0.227, 0.893			0.954, 0.621			1.460, 0.482			1.065, 0.587		
TNM staging												
I	4 (22.2)	6 (33.3)	8 (44.4)	1 (5.6)	10 (55.6)	7 (38.9)	9 (50.0)	5 (27.8)	4 (22.2)	11 (61.1)	5 (27.8)	2 (11.1)
IIA-IIC	5 (17.2)	19 (65.5)	5 (17.2)	2 (6.9)	16 (55.2)	11 (37.9)	7 (24.1)	15 (51.7)	7 (24.1)	16 (55.2)	11 (37.9)	2 (6.9)
IIIA-IIIC	6 (15.4)	23 (59.0)	10 (25.6)	4 (10.3)	19 (48.7)	16 (41.0)	12 (30.8)	18 (46.2)	9 (23.1)	17 (43.6)	17 (43.6)	5 (12.8)
IVA-IVB	2 (16.7)	6 (50.0)	4 (33.3)	2 (16.7)	5 (41.7)	5 (41.7)	2 (16.7)	7 (58.3)	3 (25.0)	8 (66.7)	3 (25.0)	3 (8.3)
*χ* ^2^, *P*	5.876, 0.437			1.598, 0.953			5.308, 0.505			3.393, 0.758		

The values in bold indicate the statistically significant difference

**Table 3:** Relationships between gene expression and clinical characteristics (Continued Table 2)

Clinical features	TUBB3			STMN1			TOP2A		
	Low	Middle	High	Low	Middle	High	Low	Middle	High
Gender									
Male	22 (28.9)	41 (53.9)	13 (17.1)	27 (35.5)	36 (47.4)	13 (17.1)	9 (11.8)	45 (59.2)	22 (28.9)
Female	13 (59.1)	8 (36.4)	1 (4.5)	7 (31.8)	12 (54.5)	3 (13.6)	5 (22.7)	11 (80.0)	6 (27.3)
*χ* ^2^, *P*	7.280, **0.026**			0.373, 0.830			1.685, 0.431		
Age									
< 57 years	21 (51.2)	16 (39.0)	4 (9.8)	19 (46.3)	19 (46.3)	3 (7.3)	8 (19.5)	24 (58.5)	9 (22.0)
> 57 years	14 (24.6)	33 (57.9)	10 (17.5)	15 (26.3)	29 (50.9)	13 (22.8)	6 (10.5)	32 (56.1)	19 (33.3)
*χ* ^2^, *P*	7.464, **0.024**			6.361, **0.042**			2.453, 0.293		
Family history									
Yes	4 (80.0)	0 (0.0)	1 (20.0)	2 (40.0)	3 (60.0)	0 (0.0)	2 (40.0)	2 (40.0)	1 (20.0)
No	31 (33.3)	49 (52.7)	13 (14.0)	32 (34.4)	45 (48.4)	16 (17.2)	12 (12.9)	54 (58.1)	27 (29.0)
*χ* ^2^, *P*	7.414, **0.025**			1.839, 0.399			2.128, 0.345		
Alcohol history									
Yes	10 (31.3)	17 (53.1)	5 (15.6)	12 (37.5)	13 (40.6)	7 (21.9)	3 (9.4)	18 (56.3)	11 (34.4)
No	25 (37.9)	32 (48.5)	9 (13.6)	22 (33.3)	35 (53.0)	9 (13.6)	11 (16.7)	38 (57.6)	17 (25.8)
	0.418, 0.812			1.681, 0.432			1.369, 0.504		
Histological type									
Tubular adenocarcinoma	29 (38.7)	34 (45.3)	12 (16.0)	23 (30.7)	38 (50.7)	14 (18.7)	10 (13.3)	40 (53.3)	25 (33.3)
Non-tubular adenocarcinoma	6 (26.1)	15 (65.2)	2 (8.7)	11 (47.8)	10 (43.5)	2 (8.7)	4 (17.4)	16 (69.6)	3 (13.0)
*χ* ^2^, *P*	2.829, 0.243			0.751, 0.253			3.551, 0.169		
Differentiation									
Undifferentiated	5 (33.3)	10 (66.7)	0 (0.0)	8 (53.3)	6 (40.0)	1 (6.7)	3 (20.0)	9 (60.0)	3 (20.0)
Poor	20 (33.3)	29 (48.3)	11 (18.3)	19 (31.7)	32 (53.3)	9 (15.0)	7 (11.7)	34 (56.7)	19 (31.7)
Moderate	9 (40.9)	10 (45.5)	3 (13.6)	7 (31.8)	10 (45.5)	5 (22.7)	4 (18.2)	13 (59.1)	5 (22.7)
Well	1 (100.0)	0 (0.0)	0 (0.0)	0 (0.0)	0 (0.0)	1 (100.0)	0 (0.0)	0 (0.0)	1 (100.0)
*χ* ^2^, *P*	8.144, 0.228			7.341, 0.290			4.279, 0.639		
Growth patterns									
Expansive	1 (50.0)	1 (50.0)	0 (0.0)	2 (100.0)	0 (0.0)	0 (0.0)	1 (50.0)	1 (50.0)	0 (0.0)
Invasive	24 (33.8)	34 (47.9)	13 (18.3)	21 (29.6)	36 (50.7)	14 (19.7)	9 (12.7)	42 (59.2)	20 (28.2)
Expansive + invasive	10 (40.0)	14 (56.0)	1 (4.0)	10 (40.0)	11 (44.0)	12 (48.0)	4 (16.0)	13 (52.0)	8 (32.0)
*χ* ^2^, *P*	4.443, 0.349			7.200, 0.126			2.694, 0.610		
Metastasis									
Yes	14 (26.9)	29 (55.8)	9 (17.3)	16 (30.8)	26 (50.0)	10 (19.2)	5 (9.6)	30 (57.7)	17 (32.7)
No	21 (45.7)	20 (43.5)	5 (10.9)	18 (39.1)	22 (47.8)	6 (13.0)	9 (19.6)	26 (56.5)	11 (23.9)
*χ* ^2^, *P*	3.843, 0.146			1.088, 0.581			2.356, 0.308		
Depth of invasion									
T1	6 (42.9)	8 (57.1)	0 (0.0)	5 (35.7)	8 (57.1)	1 (7.1)	5 (35.7)	5 (35.7)	4 (28.6)
T2	2 (40.0)	2 (40.0)	1 (20.0)	1 (20.0)	2 (40.0)	2 (40.0)	0 (0.0)	4 (80.0)	1 (20.0)
T3	1 (33.3)	1 (33.3)	1 (33.3)	0 (0.0)	1 (33.3)	2 (66.6)	0 (0.0)	1 (33.3)	2 (66.6)
T4	26 (34.2)	38 (50.0)	12 (15.8)	28 (36.8)	37 (48.7)	11 (14.5)	9 (11.8)	46 (60.5)	21 (27.6)
*χ* ^2^, *P*	5.469, 0.485			7.900, 0.246			9.263, 0.159		
Lymph node metastasis									
N0	22 (42.3)	25 (48.1)	5 (9.6)	20 (38.5)	26 (50.0)	6 (11.5)	9 (17.3)	31 (59.6)	12 (23.1)
N1	6 (18.8)	19 (59.4)	7 (21.9)	10 (31.3)	14 (43.8)	8 (25.0)	4 (12.8)	17 (53.1)	11 (34.4)
N2	5 (45.5)	4 (36.4)	2 (18.2)	4 (36.4)	5 (45.5)	2 (18.2)	0 (0.0)	8 (72.7)	3 (27.3)
Undetermined	2 (66.7)	1 (33.3)	0 (0.0)	0 (0.0)	3 (100.0)	0 (0.0)	1 (33.3)	0 (0.0)	2 (66.7)
*χ* ^2^, *P*	8.812, 0.184			6.938, 0.327			10.139, 0.119		
Distant metastasis									
M0	31 (36.0)	41 (51.2)	11 (12.8)	33 (38.4)	39 (45.3)	14 (16.3)	12 (14.0)	51 (59.3)	23 (26.7)
M1	4 (33.3)	5 (41.7)	3 (25.0)	1 (8.3	9 (75.0)	2 (16.7)	2 (16.7)	5 (41.7)	5 (41.7)
*χ* ^2^, *P*	1.148, 0.563			5.461, 0.065			1.410, 0.494		
TNM staging									
I	8 (44.4)	9 (50.0)	1 (5.6)	6 (33.3)	9 (50.0)	3 (16.7)	5 (27.8)	8 (44.4)	5 (27.8)
IIA-IIC	11 (37.9)	15 (51.7)	3 (10.3)	11 (37.9)	15 (51.7)	3 (10.3)	3 (10.3)	20 (69.0)	6 (20.7)
IIIA-IIIC	12 (30.8)	20 (51.3)	7 (17.9)	16 (41.0)	15 (38.5)	8 (20.5)	4 (10.3)	23 (59.0)	12 (30.8)
IVA-IVB	4 (33.3)	5 (41.7)	3 (25.0)	1 (8.3)	9 (75.0)	2 (16.7)	2 (16.7)	5 (41.7)	5 (41.7)
*χ* ^2^, *P*	3.629, 0.727			7.539, 0.274			5.869, 0.438		

The values in bold indicate the statistically significant difference

### UGT1A1 polymorphisms and their relationships with clinical characteristics

There were no significant differences in the genotype distribution between patients with different gender, age, family history, alcohol history, differentiation degree, growth patterns, metastasis and TNM staging (Table 4). The genotype frequencies for the UGT1A1 polymorphism at position −211 were associated with the histological type, in which patients with GG genotype showed a predisposition to developing tubular adenocarcinoma. In addition, polymorphisms at positions *28 and −3156 were correlated with the depth of invasion (both *P* = 0.023) where patients carrying 6TAA or G allele tended to have a local invasion. Combined effects for UGT1A1*28, UGT1A1-211 and UGT1A1-3156 were also performed and the results only indicated the association with the histological type (*P* = 0.043) (Table 5).

**Table 4:** UGT1A11 polymorphisms and their relationships with clinical characteristics

Clinical features	*28			−3156 GA			211GA		
	TA 6/6	TA 6/7	TA 7/7	GG	GA	AA	GG	GA	AA
Gender									
Male	64 (84.2)	10 (13.2)	2 (2.6)	64 (84.2)	10 (13.2)	2 (2.6)	49 (64.5)	23 (30.3)	4 (5.3)
Female	17 (77.3)	5 (22.7)	0 (0.0)	17 (77.3)	5 (22.7)	0 (0.0)	13 (59.1)	9 (40.9)	0 (0.0)
*χ* ^2^, *P*	2.046, 0.359			2.046, 0.359			2.674, 0.263		
Age									
< 57 years	33 (80.5)	8 (19.5)	0 (0.0)	33 (80.5)	8 (19.5)	0 (0.0)	26 (63.4)	14 (34.1)	1 (2.4)
> 57 years	48 (84.2)	7 (12.3)	2 (3.5)	48 (84.2)	7 (12.3)	2 (3.5)	36 (63.2)	18 (31.6)	3 (5.3)
*χ* ^2^, *P*	3.009, 0.222			3.009, 0.222			0.544, 0.762		
Family history									
Yes	4 (80.0)	1 (20.0)	0 (0.0)	4 (80.0)	1 (20.0)	0 (0.0)	2 (40.0)	2 (40.0)	1 (20.0)
No	77 (82.8)	14 (15.1)	2 (2.2)	77 (82.8)	14 (15.1)	2 (2.2)	60 (64.5)	30 (62.3)	3 (3.2)
*χ* ^2^, *P*	0.283, 0.868			0.283, 0.868			2.364, 0.307		
Alcohol history									
Yes	29 (90.6)	3 (9.4)	0 (0.0)	29 (90.6)	3 (9.4)	0 (0.0)	21 (65.6)	9 (28.1)	2 (6.3)
No	52 (78.8)	12 (18.2)	2 (3.0)	52 (78.8)	12 (18.2)	2 (3.0)	41 (62.1)	23 (34.8)	2 (3.0)
*χ* ^2^, *P*	3.132, 0.209			3.132, 0.209			0.861, 0.650		
Histological type									
Tubular adenocarcinoma	60 (80.0)	13 (17.3)	2 (2.7)	60 (80.0)	13 (17.3)	2 (2.7)	54 (72.0)	19 (25.3)	2 (2.7)
Non-tubular adenocarcinoma	21 (91.3)	2 (8.7)	0 (0.0)	21 (91.3)	2 (8.7)	0 (0.0)	8 (34.8)	13 (56.5)	2 (8.7)
*χ* ^2^, *P*	2.308, 0.315			2.308, 0.315			10.339, **0.006**		
Differentiation									
Undifferentiated	13 (86.7)	2 (13.3)	0 (0.0)	13 (86.7)	2 (13.3)	0 (0.0)	6 (40.0)	7 (46.7)	2 (13.3)
Poor	51 (85.0)	8 (13.3)	1 (1.7)	51 (85.0)	8 (13.3)	1 (1.7)	41 (68.3)	18 (30.0)	1 (1.7)
Moderate	17 (77.3)	4 (18.2)	1 (4.5)	17 (77.3)	4 (18.2)	1 (4.5)	14 (63.6)	7 (31.8)	1 (4.5)
Well	0 (0.0)	1 (100.0)	0 (0.0)	0 (0.0)	1 (100.0)	0 (0.0)	1 (100.0)	0 (0.0)	0 (0.0)
*χ* ^2^, *P*	5.368, 0.498			5.368, 0.498			6.642, 0.355		
Growth patterns									
Expansive	2 (100.0)	0 (0.0)	0 (0.0)	2 (100.0)	0 (0.0)	0 (0.0)	1 (50.0)	1 (50.0)	0 (0.0)
Invasive	56 (78.9)	13 (18.3)	2 (2.8)	56 (78.9)	13 (18.3)	2 (2.8)	47 (66.2)	21 (29.6)	3 (4.2)
Expansive + invasive	23 (92.0)	2 (8.0)	0 (0.0)	23 (92.0)	2 (8.0)	0 (0.0)	14 (56.0)	10 (40.0)	1 (4.0)
*χ* ^2^, *P*	3.800, 0.434			3.800, 0.434			1.295, 0.862		
*χ* ^2^, *P*	1.239, 0.538			1.239, 0.538			2.959, 0.228		
Depth of invasion									
T1	11 (78.6)	1 (7.1)	2 (14.2)	11 (78.6)	1 (7.1)	2 (14.2)	11 (78.6)	3 (21.4)	0 (0.0)
T2	2 (40.0)	3 (60.0)	0 (0.0)	2 (40.0)	3 (60.0)	0 (0.0)	4 (80.0)	1 (20.0)	0 (0.0)
T3	2 (66.7)	1 (33.3)	0 (0.0)	2 (66.7)	1 (33.3)	0 (0.0)	3 (100.0)	0 (0.0)	0 (0.0)
T4	66 (86.8)	10 (13.2)	0 (0.0)	66 (86.8)	10 (13.2)	0 (0.0)	44 (57.9)	28 (36.8)	4 (5.3)
*χ* ^2^, *P*	14.637, **0.023**			14.637, **0.023**			6.870, 0.333		
Lymph node metastasis									
N0	41 (78.8)	10 (19.2)	1 (1.9)	41 (78.8)	10 (19.2)	1 (1.9)	35 (67.3)	16 (30.8)	1 (1.9)
N1	26 (81.3)	5 (15.6)	1 (3.1)	26 (81.3)	5 (15.6)	1 (3.1)	21 (65.6)	9 (28.1)	2 (6.3)
N2	11 (100)	0 (0.0)	0 (0.0)	11 (100)	0 (0.0)	0 (0.0)	3 (27.3)	7 (63.6)	1 (9.1)
Undetermined	3 (100.0)	0 (0.0)	0 (0.0)	3 (100.0)	0 (0.0)	0 (0.0)	3 (100.0)	0 (0.0)	0 (0.0)
*χ* ^2^, *P*	6.082, 0.414			6.082, 0.414			10.126, 0.119		
Distant metastasis									
M0	69 (80.2)	15 (17.4)	2 (2.3)	69 (80.2)	15 (17.4)	2 (2.3)	57 (66.3)	25 (29.1)	4 (4.7)
M1	12 (100)	0 (0.0)	0 (0.0)	12 (100)	0 (0.0)	0 (0.0)	5 (41.7)	7 (58.3)	0 (0.0)
*χ* ^2^, *P*	4.912, 0.086			4.912, 0.086			4.485, 0.106		
TNM staging									
I	13 (72.2)	3 (16.7)	2 (11.1)	13 (72.2)	3 (16.7)	2 (11.1)	14 (77.8)	4 (22.2)	0 (0.0)
IIA-IIC	23 (79.3)	6 (20.7)	0 (0.0)	23 (79.3)	6 (20.7)	0 (0.0)	22 (75.9)	6 (20.7)	1 (3.4)
IIIA-IIIC	33 (84.6)	6 (15.4)	0 (0.0)	33 (84.6)	6 (15.4)	0 (0.0)	21 (53.8)	15 (38.5)	3 (7.7)
IVA-IVB	12 (100)	0 (0.0)	0 (0.0)	12 (100)	0 (0.0)	0 (0.0)	5 (41.7)	7 (58.3)	0 (0.0)
*χ* ^2^, *P*	11.682, 0.069			11.682, 0.069			10.771, 0.096		

The values in bold indicate the statistically significant difference

**Table 5:** UGT1A11 polymorphisms and their relationships with clinical characteristics (Continued Table 4)

Clinical features	Combination					
	TA6/6 −3156GG, 211GG	TA6/6, −3156GG, 211GA	TA6/7, −3156GA, 211GG	TA6/7, −3156GA, 211GA	TA6/6, −3156GG, 211AA	TA7/7, −3156AA, 211GG
Gender						
Male	39 (51.3)	21 (27.6)	8 (10.5)	2 (2.6)	4 (5.3)	2 (2.6)
Female	8 (36.4)	9 (40.9)	5 (22.7)	0 (0.0)	0 (0.0)	0 (0.0)
*χ* ^2^, *P*	7.516, 0.185					
Age						
< 57 years	19 (46.3)	13 (31.7)	7 (17.1)	1 (2.4)	1 (2.4)	0 (0.0)
> 57 years	28 (49.1)	17 (29.8)	6 (10.5)	1 (1.8)	3 (5.3)	2 (3.5)
*χ* ^2^, *P*	3.541, 0.617					
Family history						
Yes	1 (20.0)	2 (40.0)	1 (20.0)	0 (0.0)	1 (20.0)	0 (0.0)
No	46 (49.5)	28 (30.1)	12 (12.9)	2 (2.2)	3 (3.2)	2 (2.2)
*χ* ^2^, *P*	3.571, 0.613					
Alcohol history						
Yes	18 (56.3)	9 (28.1)	3 (9.4)	0 (0.0)	2 (6.3)	0 (0.0)
No	29 (43.9)	21 (31.8)	10 (15.2)	2 (3.0)	2 (3.0)	2 (3.0)
*χ* ^2^, *P*	7.516, 0.185					
Histological type						
Tubular adenocarcinoma	40 (53.3)	18 (24.0)	12 (16.0)	1 (1.3)	2 (2.7)	2 (2.7)
Non-tubular adenocarcinoma	7 (30.4)	12 (52.2)	1 (4.3)	1 (4.3)	2 (8.7)	0 (0.0)
*χ* ^2^, *P*	11.488, **0.043**					
Differentiation						
Undifferentiated	5 (33.3)	6 (40.0)	1 (6.7)	1 (6.7)	2 (13.3)	0 (0.0)
Low	32 (53.3)	18 (30.0)	8 (13.3)	0 (0.0)	1 (1.7)	1 (1.7)
Moderate	10 (45.5)	6 (27.3)	3 (13.6)	1 (4.5)	1 (4.5)	1 (4.5)
High	0 (0.0)	0 (0.0)	1 (100.0)	0 (0.0)	0 (0.0)	0 (0.0)
*χ* ^2^, *P*	14.419, 0.494					
Growth patterns						
Expansive	1 (50.0)	1 (50.0)	0 (0.0)	0 (0.0)	0 (0.0)	0 (0.0)
Invasive	34 (47.9)	19 (26.8)	11 (15.5)	2 (2.8)	3 (4.2)	2 (2.8)
Expansive + invasive	12 (48.0)	10 (40.0)	2 (8.0)	0 (0.0)	1 (4.0)	0 (0.0)
*χ* ^2^, *P*	5.361, 0.866					
Metastasis						
Yes	23 (44.2)	19 (36.5)	5 (9.6)	1 (1.9)	3 (5.8)	1 (1.9)
No	24 (52.2)	11 (23.9)	8 (17.4)	1 (2.2)	1 (2.2)	1 (2.2)
*χ* ^2^, *P*	3.558, 0.615					
Depth of invasion						
T1	8 (57.1)	3 (21.4)	1 (7.1)	0 (0.0)	0 (0.0)	2 (14.3)
T2	1 (20.0)	1 (20.0)	3 (60.0)	0 (0.0)	0 (0.0)	0 (0.0)
T3	2 (66.7)	0 (0.0)	1 (33.3)	0 (0.0)	0 (0.0)	0 (0.0)
T4	36 (47.4)	26 (34.2)	8 (10.5)	2 (2.6)	4 (5.3)	0 (0.0)
*χ* ^2^, *P*	21.061, 0.135					
Lymph node metastasis						
N0	25 (48.1)	15 (28.8)	9 (17.3)	1 (1.9)	1 (1.9)	1 (1.9)
N1	16 (50.0)	8 (25.0)	4 (12.5)	1 (3.1)	2 (6.3)	1 (3.1)
N2	3 (27.3)	7 (63.6)	0 (0.0)	0 (0.0)	1 (9.1)	0 (0.0)
Undetermined	3 (100.0)	0 (0.0)	0 (0.0)	0 (0.0)	0 (0.0)	0 (0.0)
*χ* ^2^, *P*	15.280, 0.431					
Distant metastasis						
M0	42 (48.8)	23 (26.7)	13 (15.1)	2 (2.3)	4 (4.7)	2 (2.3)
M1	5 (41.7)	7 (58.3)	0 (0.0)	0 (0.0)	0 (0.0)	0 (0.0)
*χ* ^2^, *P*	8.417, 0.135					
TNM staging						
I	9 (50.0)	4 (22.2)	3 (16.7)	0 (0.0)	0 (0.0)	2 (11.1)
IIA-IIC	16 (55.2)	6 (20.7)	6 (20.7)	0 (0.0)	1 (3.4)	0 (0.0)
IIIA-IIIC	17 (43.6)	13 (33.3)	4 (10.3)	2 (5.1)	3 (7.7)	0 (0.0)
IVA-IVB	5 (41.7)	7 (58.3)	0 (0.0)	0 (2.6)	0 (5.3)	0 (0.0)
*χ* ^2^, *P*	23.131, 0.081					

The values in bold indicate the statistically significant difference

## Discussion

Individualized therapeutic regimen is a recently intensively pursued approach for targeting diseases at the molecular level, in which the search for biomarkers was considered the first and most important [28, 29]. Therefore, the present study was to investigate whether the genes ERCC1, BRCA1, TYMS, RRM1, TUBB3, STMN1 and TOP2A are underlying biomarkers for patients with gastric cancer, which, to our knowledge, has not been performed. However, our results showed that only the expression levels of TYMS, TUBB3 and STMN1 were significantly associated with the clinical characteristics of age, gender and family history of gastric cancer, but not with differentiation degree, growth patterns, metastasis and TNM staging in patients with gastric cancer. No clinical characteristics were correlated with the expressions of ERCC1, BRCA1, RRM1 and TOP2A. These findings seemed not to be fully in accordance with the studies of other cancers [3–8], suggesting the difference among different cancers.

TUBB3 is one of the major components of microtubules (a basic constructive unit of spindle) to be involved in cell division, thus promoting the possibility of malignant growth and metastases [30]. As expected, TUBB3 is shown to be higher expressed in various cancers than that in healthy control, including gastric cancer (31.6 ± 17.8 ng/mL vs. 16.9 ± 3.8 ng/mL, *p* < 0.001) [31]. To reduce the expression of TUBB3 and improve survival of cancer patients, several anti-microtubule chemotherapeutic agents (i.e. paclitaxel, capecitabine, carboplatin) were suggested and the studies demonstrated that the patients were more sensitive to these treatments when TUBB3 mRNA expression was low enough [31–34]. In this study, we found TUBB3 was relatively lower expressed in the females (*P* = 0.026), patients with age < 57 years (*P* = 0.024) and family history of gastric cancer (*P* = 0.025), suggesting the above anti-microtubule chemotherapeutic agents should be recommended for these patients to obtain more benefits.

STMN1 encodes a regulatory protein that participates in assembly and disassembly of the mitotic spindle, facilitating the achievement of mitosis process and uncontrolled proliferation of malignant tumor cells [35]. Hence, STMN1 should be also highly expressed in gastric cancer, which has been demonstrated by the study of Kang et al. who report an up-regulated expression of STMN1 in 80 and 56% primary gastric adenocarcinomas at protein and mRNA levels, respectively [36]. Knock-down of STMN1 using siRNA inhibits the proliferation, migration and invasion of gastric cancer cells and slows the growth of xenografts in nude mice [37–39]. Although STMN1 over-expression has been reported to be positively correlated with lymph node metastasis and TNM staging [36, 37, 40], no association was proved in our study, which may be attributed to small sample size and different detection methods. Moreover, studies imply that cancer patients with low expression of STMN1 mRNA will have a favorable clinical efficacy after being treated with Taxol regimens [41, 42]. Silencing STMN1 enhances the sensitivity of gastric cancer cells to docetaxel, with the resistance index reducing to 3.41 [13]. In this study, we found STMN1 was relatively lower expressed in the patients with age < 57 years (*P* = 0.042), suggesting Taxol and docetaxel may be a more appropriate treatment for patients aged < 57 years.

TYMS is a central enzyme in the folate metabolic pathway, which catalyzes the conversion of deoxyuridine monophosphate to deoxythymidine monophosphate, thereby maintaining the dTMP (thymidine-5-prime monophosphate) pool critical for DNA replication and repair. Tumor cells with higher TYMS expression exhibited higher proliferative and metastatic activities due to its accelerated effect on the DNA replication [43, 44]. Thus, scholars suggest TYMS may be a potential biomarker for gastric cancer and response rates for the antifolate cytotoxic chemotherapy (i.e. raltitrexed, fluoropyrimidine) may be higher in patients with low expression of TYMS [45, 46].

Moreover, we also investigated whether the genetic variation in UGT1A1 gene was related with the clinical characteristics of gastric cancer, which, to our knowledge, has also not been performed. The results showed that patients carrying G allele at −211 were predisposed to developing tubular adenocarcinoma, while patients carrying 6TAA or G allele respectively at *28 and −3156 tended to have a local invasion. Our results are similar to previous studies because the -211GG, −211GA, *28 6TAA/6TAA, *28 6TAA/7TAA, −3156 GG or −3156 GA contribute to high expression of UGT1A1 gene, which causes high levels of glucuronidation and then reduces serum bilirubin levels. Lower bilirubin did not exert protective effects against cancer development and progression by inhibiting cellular damage induced by oxidative stress [26, 47]. Therefore, bilirubin levels should be moderately elevated to improve the overall survival of these patients [48].

Our study had some limitations. Firstly, this was a single institutional study and sample size was not enough large, which may result in different conclusions with previous studies. Secondly, the gene expression was only detected using the multiplex branched DNA liquidchip technology, but not confirmed by immunohistochemistry with normal stomach tissues or para-carcinoma tissues as control. However, we believe our study may be important because the expression level (low, middle, high) detected by the MBL technology may be more effective to guide the prognosis and individualized chemotherapy as previously described [3–8]. The studies about comparison with normal stomach tissues or para-carcinoma tissues only indicate the upregulation or downregulation, but not further classify the degree. Furthermore, the expression of 7 genes in comparison with normal stomach or adjacent normal has been independently investigated previously [9–15]. Thirdly, gastric cancer samples were only categorized according to the WHO’s, but not by the Lauren’s histological classification system in our hospital. However, recent studies indicate there is a high concordance between two systems [49, 50]. Fourthly, chemotherapy and follow up results had not been collected completely, although they have been performed in our center. This resulted in the lack of analysis on cost effectiveness ratio, which may be the most concern for patients. Nevertheless, we believe the average cost effectiveness ratio may be lower because our detection can guide the individualized chemotherapy arrangement, which may be beneficial to 1) improve the efficacy and prolong life; 2) reduce the side effect due to the inappropriate chemotherapy drugs, and enhance the quality of life; 3) prevent a waste of money due to the repeated attempts of chemotherapy drugs/scheme. In summary, further investigation is still essential to confirm the biomarker role of TYMS, TUBB3, STMN1 and UGT1A1 by a multicenter clinical study with large sample size, more classification systems, addition of control samples, immunohistochemistry confirmation and chemotherapy results.

## Conclusions

Our findings suggest UGT1A1 polymorphisms may be useful to screen the risk population of gastric cancer and schedule the appropriate pretreatment to improve the overall survival, while TYMS, TUBB3 and STMN1 may be potential biomarkers for prognosis and chemotherapy guidance.

## Additional file

Additional file 1: Raw data of patients. The general characteristics, gene expressions and polymorphisms of each patient are available. (XLS 54 kb)

## Abbreviations

BRCA1: Breast cancer susceptibility gene breast cancer 1
dTMP: Thymidine-5-prime monophosphate
ERCC1: Excision repair cross complementing 1
FFPE: Formalin fixed paraffin embedded
MBL: Multiplex branched DNA liquidchip
RRM1: Ribonucleotide reductase M1
STMN1: Stathmin1
TOP2A: Topoisomerase IIα
TUBB3: β-tubulin III
TYMS: Thymidylate synthetase
UGTs: UDP-glucuronosyltransferases
